# Prognostic value of tumor mutations in radically treated locally advanced non-small cell lung cancer patients

**DOI:** 10.18632/oncotarget.15966

**Published:** 2017-03-07

**Authors:** Angela Boros, Ludovic Lacroix, Benjamin Lacas, Julien Adam, Jean-Pierre Pignon, Caroline Caramella, David Planchard, Vincent de Montpreville, Eric Deutsch, Antonin Levy, Benjamin Besse, Cécile Le Pechoux

**Affiliations:** ^1^ Radiation Oncology Department, Gustave Roussy, Villejuif, France; ^2^ Biopathology Department, Gustave Roussy, Villejuif, France; ^3^ Biostatistics and Epidemiolgy Unit, Villejuif, France; ^4^ Imaging Department, Gustave Roussy, Villejuif, France; ^5^ Medical Oncology Department, Gustave Roussy, Villejuif, France; ^6^ Pathology Department, Marie Lannelongue, Le Plessis Robinson, France; ^7^ Paris-Sud University, DHU TORINO, Paris, France; ^8^ Paris-Saclay University, Paris, France; ^9^ INSERM U1030, Villejuif, France; ^10^ INSERM U1018, Villejuif, France

**Keywords:** locally advanced, non-small cell lung cancer, mutation, prognostic, chemotherapy

## Abstract

**Introduction:**

Chemo-radiation is standard treatment in locally advanced non-small cell lung cancers (NSCLC). The prognostic value of mutations has been poorly explored in this population.

**RESULTS:**

Clinical data were collected from 190 patients and mutational profiles were obtained in 78 of them; 58 (74%) were males, 31 (40%) current smokers, 47/31 stage IIIA/IIIB and 40 (51%) adenocarcinoma. The following mutations were identified: *EGFR* 12% (9/78), *KRAS* 15% (12/78), *BRAF* 5% (3/65), *PI3KCA* 2% (1/57), *NRAS* 3% (1/32), and ALK+ (FISH) 4% (2/51). *HER2* was not detected. Median follow-up was 3.1 years. Overall survival was evaluated by group; no significant differences were identified in median overall survival (*p* = 0.21), with 29.4 months for the *EGFR/ALK* group (*n* = 11), 12.8 months for other mutations (*n* = 17), and 23.4 months for wild-type (*n* = 50). The *EGFR/ALK* and other mutations groups had poorer median progression-free survival (9.6 and 6.0 months) compared to the wild-type group (12.0 months; multivariate hazard ratio 2.0 [95% CI, 0.9–4.2] and 2.8 [95% CI, 1.5–5.2] respectively, *p* = 0.003).

**Materials and Methods:**

We retrospectively reviewed all patients receiving radical treatment for locally advanced NSCLC in a single institution between January 2002 and June 2013. Next generation sequencing was performed on DNA from paraffin-embedded tissue. *ALK* rearrangements were detected by immunohistochemistry and/or FISH. Mutational prognostic value for Kaplan-Meier survival parameters was determined by log-rank tests and Cox proportional hazards models.

**Conclusions:**

Selected gene alterations may be associated with poorer progression-free survival in locally advanced radically treated NSCLC and their prognostic and/or predictive value merits further evaluation in a larger population.

## INTRODUCTION

Molecular profiling has become a standard procedure in advanced non-small cell lung cancer (NSCLC). Specific tyrosine kinase oncogenic activation, especially epidermal growth factor receptor (*EGFR*) mutations or rearrangement of the anaplastic lymphoma kinase (*ALK*) gene, led to the development of targeted molecular inhibitors and a specific therapeutic strategy in this patient subgroup [1, 2, 3]. Uncommon NSCLC mutations such as *HER2, BRAF* and *PI3KCA* might also be relevant targets [4, 5]. In advanced NSCLC, the presence of an *EGFR* or the *BRAF V600* mutation confers a more favorable prognosis while the *KRAS* mutation is associated with worse outcomes [6, 7].

The standard of care in inoperable stage III NSCLC patients with good performance status is concomitant chemo-radiation, conferring an absolute benefit of 4.5% in terms of 5-year survival compared to sequential chemo-radiation [8] for patients with no or limited comorbidities and adequate organ function [9, 10]. There is no standard chemotherapy regimen, but platinum-based doublets are associated with better progression-free survival (PFS) [8]. While treatment choice guided by gene alterations is a widely-used strategy in stage IV NSCLC, molecular abnormalities rarely influence treatment choices in locally advanced NSCLC patients and furthermore, the outcome according to gene alterations is unknown. This study was designed to explore the prognostic value of specific gene alterations in locally advanced NSCLC patients, in light of moving towards personalized treatment strategies in this patient population.

## RESULTS

### Patients and treatments

**Figure 1:**
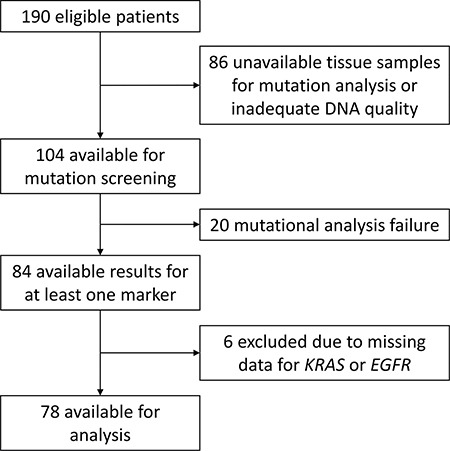
Patient flow chart

Among the 190 eligible patients, 84 patients (44%) had available data for at least one marker. Six were excluded because of missing data for *KRAS* and/or *EGFR* (Figure 1). The populations with and without available mutation data were well balanced, except for stage and diabetes (more patients with stage IIIA and diabetes in the population with data (Table 1).

**Table 1:** Patient and treatment characteristics in patients with and without mutation profiling data

	Mutation data (*N* = 78)		No mutation data (*N* = 112)		*p*-value (χ^2^)
	*N*	%	*N*	%	
**Sex**					0.40
Female	20	*26*	35	*31*	
Male	58	*74*	77	*69*	
**Age (years)**					0.27
< 60	44	*56*	54	*48*	
≥ 60	34	*44*	58	*52*	
Median[range]	57.9 [30.7 ; 88.9]		60.6 [34.4 ; 85.0]		
**Performance status**					0.08
0	38	*49*	69	*62*	
≥ 1	40	*51*	43	*38*	
**Histology**					0.10
Adenocarcinoma	40	*51*	44	*39*	
Other*	38	*49*	68	*61*	
**T-stage**					0.60
T0–T2	35	*45*	46	*41*	
T3–T4	43	*55*	66	*59*	
**N-stage**					0.53
N0–N1	13	*17*	15	*13*	
N2–N3	65	*83*	97	*87*	
**Stage**					0.001
IIIA	47	*60*	41	*37*	
IIIB	31	*40*	71	*63*	
**Smoking status**					0.16
Current smoker	31	*40*	56	*50*	
Never or former smoker	47	*60*	56	*50*	
**Diabetes**					0.04
No	68	*87*	107	*96*	
Yes	10	*13*	5	*4*	
**Radiotherapy dose**					0.78
< 66 Gy	27	*35*	41	*37*	
≥ 66 Gy	51	*65*	71	*63*	
**Thoracic surgery**					0.06
No	60	*77*	98	*87*	
Yes	18	*23*	14	*13*	

* Includes large cell carcinoma, mixed cell carcinoma, neuroendocrine markers, squamous cell carcinoma, undifferentiated tumor.

Among the 78 patients with available mutation data, 58 (74%) were male, 31 (40%) were current smokers, 40 (51%) had adenocarcinoma, 47 patients (60%) had stage IIIA, and 31 patients (40%) had IIIB. An initial positron emission tomography scan (PET-CT) was performed on 66 patients (85%). Most patients (62, 79%) had conformal radiotherapy, with a median dose of 66 Gy in 33 fractions (f) and a median 48 days overall treatment time; three patients had moderate hypofractionated radiotherapy (2.5 Gy/f) and one had split-course radiotherapy (55 Gy/20f). Eighteen patients (23%) had a history of thoracic surgery, either with curative intent or at relapse. Twelve patients (15%) had undergone curative surgery and received adjuvant radiotherapy. Five patients (6%) had surgery without adjuvant treatment, undergoing radiotherapy for mediastinal relapse. One patient underwent surgery for local relapse. Platinum-based chemotherapy was concomitantly administered to 50 patients (64%), as induction/consolidation treatment to 67 patients (86%), and 7 patients did not receive chemotherapy, mainly because of poor performance status or age over 75 years. The most frequently administered chemotherapy regimen was cisplatin/vinorelbine (33 patients, 42%). Median follow-up was 3.1 years.

### Mutation profiling

Of the 78 patients analyzed, 28 (36%) had a mutation. One had both an *ALK* rearrangement and a *KRAS* mutation, and was considered *ALK* wild-type due to a higher prevalence of *KRAS* mutation in reported NSCLC population. Another patient was *KRAS* mutant but was missing data for *EGFR*. Since *KRAS* and *EGFR* mutations were mostly mutually exclusive, this patient was considered *EGFR* wild-type.

Mutations identified among the 78 patients (before considering missing data as wild-type) were *EGFR* 12% (9/78), *KRAS* 15% (12/78), *BRAF* 5% (3/65), *PI3KCA* 2% (1/57), *NRAS* 3% (1/32) and *ALK+* (by FISH) in 4% (2/51). *HER2* was not detected (Supplementary Table 1). Given the small patient numbers, the population was divided into three groups for prognostic analyses: 50 (64%) in the wild-type group, 11 (14%) in the *EGFR/ALK* group, and 17 (22%) in the other mutation group.

### Survival and prognostic factors

**Figure 2:**
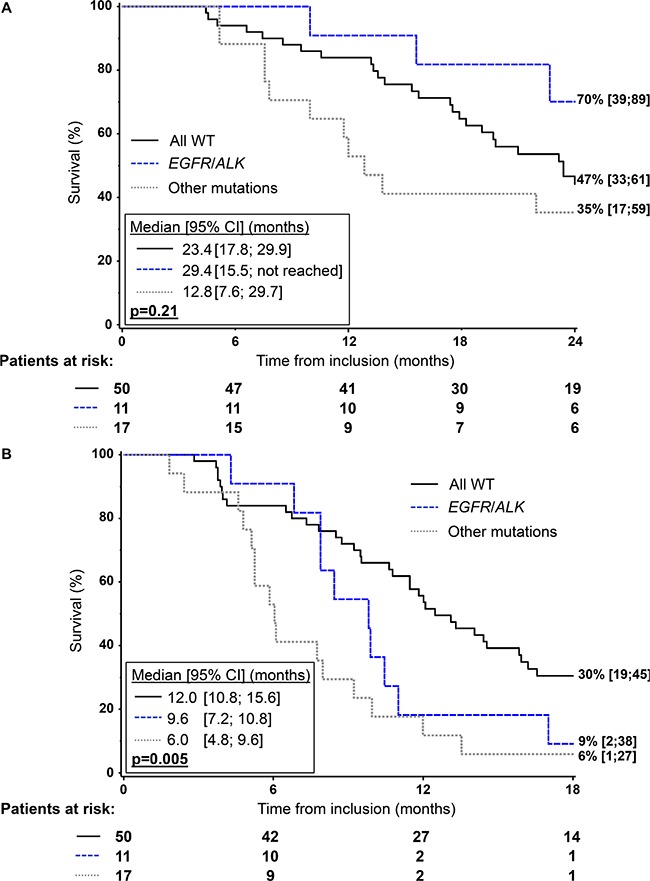
Kaplan-Meier analysis of overall survival (**A**) and progression-free survival (**B**) according to mutation status in the 78 patients with mutation profiling.

Median survival was 23.4 months [95% confidence interval (CI) 18.2–29.1]. OS was not significantly different (*p* = 0.21) between the three groups: 29.4 months [95% CI 15.5 - not reached] for *EGFR/ALK*, 12.8 months [95% CI 7.6–29.7] for other mutations and 23.4 months [95% CI 17.8–29.9] for wild-type (Figure 2A). Of the 11 patients in the *EGFR/ALK* group, 7 had received targeted agents at recurrence. In the multivariate analysis, OS was not significantly different (*p* = 0.26) between the three groups, with a hazard ratio (HR) of 0.8 [95% CI 0.3–2.0] and 1.6 [95% CI 0.8–3.2] in the *EGFR/ALK* and other mutation groups, compared to the wild-type group, respectively (Table 2). Having a PET-scan was the only factor significantly associated with OS (HR = 0.3 [95% CI 0.1–0.6], *p* = 0.002). A non-significant trend for better survival was associated with performance status 0 vs. ≥ 1 (HR = 1.7 [95% CI 0.9–2.9]; *p* = 0.08).

**Table 2:** Univariable and multivariable (Cox) analyses of prognostic factors for overall survival in the mutation analysis group

	No. deaths /No. patients	Univariable	Multivariable	
		*p*-value	HR [95% CI]	*p*-value
**Mutation group**		0.22		0.26
All wildtype	34/50		Ref	
*EGFR/ALK+*	6/11		0.8 [0.3 ; 2.0]	
Other mutation	13/17		1.6 [0.8 ; 3.2]	
**Performance status**		0.08		0.08
0	23/38		Ref	
≥ 1	30/40		1.7 [0.9 ; 2.9]	
**Stage**		0.17		0.10
IIIA	29/47		Ref	
IIIB	24/31		1.7 [0.9 ; 3.0]	
**Radiotherapy dose**		0.55		
< 66 Gy	20/27			
≥ 66 Gy	33/51			
**Thoracic surgery**		0.16		0.20
No	42/60		Ref	
Yes	11/18		0.6 [0.3 ; 1.3]	
**PET scan**		0.05		0.002
No	11/12		Ref	
Yes	42/66		0.3 [0.1 ; 0.6]	

HR = Hazard ratio; CI = Confidence interval; Ref = reference group.

Variables are included in the multivariable Cox model only if univariable *p*-value < 0.20, except for mutation group since it is the studied variable.

The *EGFR/ALK* and other mutation groups had significantly poorer PFS (median: 9.6 months [95% CI 7.2–10.8] and 6.0 months [95% CI 4.8–9.6], respectively) than the wild-type group (median: 12.0 months [95% CI 10.8–15.6]; *p* = 0.005) (Figure 2B). In the multivariate analysis, HRs were 2.0 [95% CI 0.9–4.2] for *EGFR/ALK* and 2.8 [1.5; 5.2] for other mutation group; *p* = 0.003) compared to wild-type (Table 3).

**Table 3:** Multivariable Cox analysis of prognostic factors for progression-free survival in the mutation analysis group

	No. events/No. patients	HR [95% CI]	*p*-value
**Mutation groups**			0.003
All wild-type	41/50	Ref	
*EGFR/ALK+*	10/11	2.0 [0.9 ; 4.2]	
Other mutation	16/17	2.8 [1.5 ; 5.2]	
**Performance status**			0.12
0	32/38	Ref	
≥ 1	35/40	1.5 [0.9 ; 2.5]	
**Stage**			0.21
IIIA	39/47	Ref	
IIIB	28/31	1.4 [0.8 ; 2.4]	
**Thoracic surgery**			0.82
No	52/60	Ref	
Yes	15/18	0.9 [0.5 ; 1.8]	
**PET scan**			0.14
No	11/12	Ref	
Yes	56/66	0.6 [0.3 ; 1.2]	

HR = Hazard ratio; CI = Confidence interval; Ref = reference group.

Taking into consideration the small number of patients in each mutational group, no difference was observed between the three groups regarding the pattern of failure of locoregional, metastatic, or locoregional and metastatic (Supplementary Table 2). Brain metastases were observed in 16 patients (21%), 10 in the wild-type group, three in the *EGFR/ALK* group and three in the other mutation group (Fisher test, *p* = 0.84).

## DISCUSSION

In recent years, mutation profiling has become increasingly important for defining the treatment strategy in stage IV NSCLC. As a result, improved OS has been observed in the subgroup of metastatic patients with targetable driver mutations such as *EGFR* mutations or *ALK* rearrangements. The goal of this retrospective study was to evaluate whether molecular profiling has any role in the outcome for locally advanced NSCLC patients. We found that the presence of specific gene alterations was associated with poorer PFS; a significant HR superior or equal to 2 in multivariate analysis was observed between the *EGFR/ALK* and wild-type groups, and other mutation and wild type groups. The corresponding median PFS were 9.6 months in the *EGFR/ALK* group, 6.0 months in the other mutation group and 12.0 months in the wild-type group. However when considering OS, there was no significant difference between the three groups, although there was a trend for improved OS in the *EGFR/ALK* group as many of these patients with actionable mutations received TKIs upon failure. While OS has historically been considered the most relevant and robust clinical endpoint, a recent large study supports PFS as a valid surrogate for OS in trials evaluating chemo-radiation in locally advanced lung cancer [11]. However, the efficacy observed with TKIs administered at relapse to patients with actionable driver mutations versus those without, supports the validity of PFS as an OS surrogate.

The prognostic value of mutations in NSCLC has been little studied in stage III patients, but has been the object of studies both in earlier and more advanced NSCLC. In a meta-analysis of surgically resected TKI-naïve NSCLC patients, including stage I to IIIA, the presence of *EGFR* mutation was not prognostic [12]. This should be interpreted with caution considering the heterogeneity in patient selection, the adjustment or not for other prognostic factors, the follow-up and techniques used to detect *EGFR* mutations. Furthermore the presence of mutations is frequently observed in patients with better clinico-pathologic features such as never smokers or female gender [13]. A pooled analysis from three adjuvant chemotherapy trials of 295 patients with lung adenocarcinoma harboring *KRAS* wild-type and known *EGFR* status did not find a correlation between *EGFR* mutational status and prognosis or predictive value for OS and PFS [14].

Among patients with more advanced disease, *EGFR* mutations are an important predictive biomarker of TKI benefit in terms of PFS for all settings, front-line, maintenance, and second-line or subsequent therapy. Some published data suggest that EGFR mutations may be a positive prognostic factor irrespective of treatment for patients with advanced disease [15]. However, data on the prognostic value of *EGFR* mutations are scarce, lack data for untreated controls and generally have small sample sizes, meaning robust conclusions cannot be drawn [16, 17]. Similarly, the prognostic value of *ALK* rearrangement is not clearly established as contradictory results have been published [18, 19, 20]. To our knowledge, the current study is among the first evaluating the prognostic value of these mutations in patients with stage III treated by definitive radiotherapy treatment.

The other mutation group of our study, which was mainly *KRAS*, had a worse PFS than wild-type, with a significant HR of 2.8 in multivariate analysis. The negative prognostic impact of *KRAS* mutations in NSCLC suggested by two systematic reviews [21, 22] was not confirmed in randomized trials evaluating adjuvant strategies in resected patients [23]. The pooled analysis of four large trials comparing adjuvant chemotherapy to observation did not find a significant prognostic value of *KRAS* mutation status among the 763 patients in the observation arm. Even if the type of mutation (codon 12 or 13) is not prognostic, further studies are needed to evaluate its predictive role [24]. More recently, studies have suggested that the prognostic impact of *KRAS* mutation could be related to the presence of concurrent mutations such as *STK11* that could define an aggressive subtype of lung cancer [25]. The prognostic value of other mutations as *PI3KCA, BRAF, NRAS*, or *HER2* in NSCLC is poorly described, mostly because of their low incidence.

Another interesting observation, for which data in stage III patients are rare, is that patient characteristics and the distribution of mutations were similar to those observed in Caucasian populations with more advanced disease. The French National Cancer Institute established a national network in 2006, providing a routine panel of biomarkers, including *EGFR, KRAS, BRAF, PI3KCA* and *HER2* mutations and *ALK* rearrangements in patients with advanced NSCLC. Clinical characteristics and outcome for almost 18,000 patients was studied, [26] giving a distribution of *EGFR* mutations (11%), *ALK* rearrangements (5%), *PI3KCA* (2%), *BRAF* (2%) and *HER2* mutations (1%). The distribution in our study was similar, except for *KRAS* mutations which seemed to be more frequent in advanced disease (29%), probably due to the higher incidence of adenocarcinoma [27].

The presence of specific gene alterations seems prognostic here, but no firm conclusions can be drawn from this small retrospective study. It nonetheless raises the question of the underlying mechanism explaining these findings. Could certain gene alterations be predictive of radio-sensitivity or radio-resistance? In this case, a different pattern of failure could be identified for each gene alteration, however no significant differences in terms of patterns of failure were observed across the three groups. OS was not significantly different between the three groups, with possibly better OS in the *EGFR/ALK* group, the latter likely driven by subsequent TKIs at relapse. A meta-analysis showed improvement in PFS and overall response rate in advanced NSCLC receiving EGFR TKIs compared to chemotherapy, with no benefit in OS, probably because of the crossover between the two arms [28]. Knowledge of *EGFR* status is therefore recommended for patient selection before EGFR-TKI therapy [29, 30].

A difference in terms of local relapse suggestive of a direct relationship with an underlying mechanism of radio-resistance was not observed in our series, or for distance relapse, although results should be interpreted with caution due to the small sample size. The radio-resistance hypothesis was highlighted in a few studies suggesting the role of *EGFR* or *KRAS* mutation as radio-resistance markers and the use of therapeutics targeting the EGFR to overcome this resistance [31, 32]. Nevertheless, *EGFR* mutations as well as *ALK* rearranged NSCLC were described as possible markers of radiosensitivity [33, 34]. Promising results have been observed in patients with radiosensitive oncogene driver mutations, particularly with oligoprogressive disease, eligible for local treatment of selected metastases [35]. A similar study evaluating crizotinib in patients with stage IV *ALK+* NSCLC and oligoprogressive extracranial disease who received local therapy, showed a PFS benefit [36]. The question of whether certain gene alterations could be predictive of radio-sensitivity or radio-resistance needs to be further explored for advanced stage and particularly for stage III NSCLC.

Progress in treatment of stage III disease is challenging, requiring both local and systemic optimal therapy. Recent phase III trials have shown better results with 2-year survival rates surpassing 50%, and median survival around 24 months [37, 38, 39]. Improved survival compared to historical series may be due to better selection and better treatment. In our study, as in most recently published phase III studies with stage III NSCLC, most patients underwent PET-CT. Treatment modalities explored in phase III trials concern different chemoradiation schedules, radiotherapy dose escalation, implementation of TKIs such as cetuximab, as well as surgery or no surgery [37, 38, 39]. Several treatment options are thus available for stage III patients, combining chemotherapy, radiotherapy and/or surgery, as well as other promising strategies that need to be further explored [40, 41, 42].

So what then is the best treatment option for an individual patient with stage III heterogeneous disease? Selected gene alterations may be associated with poorer PFS in locally advanced NSCLC patients, notably with respect to EGFR. Further studies in large populations are warranted to define the role of molecular determinants in these patients, their prognostic and/or predictive value, not only in terms of systemic treatment but also local treatment. If these results are confirmed, the strategy for locoregional management of stage III NSCLC could also be personalized based on the patients’ molecular profile, a therapeutic management strategy which is already routine practice in stage IV NSCLC patients.

## MATERIALS AND METHODS

### Eligibility

Clinical data were reviewed from all consecutive patients in our institution who received chemo-radiation, exclusive radiotherapy or tri-modality treatment with a curative intent for primary or locally recurrent stage III NSCLC between January 2002 and June 2013. An electronic search was performed using a clinical data management system, a radiotherapy data management system and the MSN database (Identification of Marker of Primary or Acquired Resistance to Anti Tumorous Treatment; NCT02105168). Of the 356 patients screened, 190 were eligible. Reasons for exclusion were palliative radiotherapy, other histology, metastatic disease at diagnosis, or death before radiotherapy. Stage III disease was defined retrospectively according to IASLC/UICC7 [43] and histologic subtype was classified according to the WHO version for lung cancer [44]. Progression was defined as the first documented radiologic evidence according to RECIST v1.1.

### Mutational analysis

Formalin-fixed, paraffin-embedded tissue blocks were collected and the presence of adequate tumor tissue was verified by the study pathologist. Analyses were performed for all samples and were considered as contributive only if more than 15% tumor cells were present. Mutational status was determined using next generation sequencing based on Ion Torrent with AmpliSeq Cancer Hotspot panel v2 (CHP2) panel as previously described [45]. In our hands, this approach offers a detection limit of 5% with a high specificity and sensitivity as previously reported with equivalent approaches [46]. *ALK* rearrangements were screened with immunohistochemistry and confirmed by fluorescence *in situ* hybridization (FISH).

### Statistical analyses

Seven markers were studied; if the mutation rate was less than 5% in the dataset, missing values were considered wild-type. Analyses were performed in three groups: wild-type (no mutations in any of the seven markers), *EGFR/ALK*, and “other mutation” (*KRAS/NRAS/BRAF/PIK3CA/HER2*). A mutant was defined as at least one mutated marker.

Patient characteristics were compared with a Chi^2^ test. Survival parameters were analyzed with the Kaplan-Meier method and mutational prognostic value was determined using the log-rank test and Cox proportional hazards models for which relevant variables were tested in a univariate analysis on overall survival (OS). If *p* ≤ 0.20, variables were added to the multivariate Cox model. The model was adjusted on ECOG performance status (0, ≥ 1), stage (IIIA, IIIB), thoracic surgery (yes, no) and initial PET-scan (yes, no). The same model was used for progression-free survival (PFS). Median follow-up was estimated by the Schemper method [47]. Analyses were performed with SAS (version 9.3). All *p*-values were two-sided.

## SUPPLEMENTARY MATERIALS TABLES


