# Supplementary material for: Prognostic value of tumor mutations in radically treated locally advanced non-small cell lung cancer patients
Source: Oncotarget. 2017 Mar 7;8(15):25189–99. doi: 10.18632/oncotarget.15966 (PMC5421921; doi:10.18632/oncotarget.15966)
Supplement: Supplementary file 1 [file oncotarget-08-25189-s001.pdf]

Prognostic value of tumor mutations in radically treated locally advanced non-small cell lung cancer patients

Supplementary Materials

Supplementary Table 1: Mutational marker reported in the mutational profiling group

	Mutation groups (N = 78)	
	N	**%**
EGFR		
Wild-type	69	*88*
Mutant	9	*12*
KRAS		
Wild-type	66	*85*
Mutant	12	*15*
BRAF		
Wild-type	62	*79*
Mutant	3	*4*
Missing*	13	*17*
PIK3CA		
Wild-type	56	*72*
Mutant	1	*1*
Missing*	21	*27*
HER2		
Wild-type	62	*79*
Mutant	0	-
Missing*	16	*21*
NRAS		
Wild-type	31	*40*
Mutant	1	*1*
Missing*	46	*59*
ALK (FISH test)		
Positive	2	*3*
Negative	49	*63*
Missing*	27	*35*

*When the mutation rate was < 5% in the dataset, missing values were considered as wild-type.

Supplementary Table 2: Type of failure description according to mutation group

	All wild-type		EGFR/ALK		Other mutation	
	N	**%**	***N***	**%**	***N***	**%**
Locoregional	12	*24*	2	*18*	2	*12*
Metastatic	11	*22*	4	*36*	5	*29*
Locoregional + metastatic*	15	*30*	4	*36*	6	*35*
Death without progression	3	*6*	0	-	3	*18*
Alive without progression	9	*18*	1	*9*	1	*6*

*Apparition of metastatic relapse less than 3 months after the diagnosis of loco-regional relapse.
